# Migration intentions among nursing students in a low-middle-income country

**DOI:** 10.1186/s12912-024-02180-9

**Published:** 2024-07-18

**Authors:** Cletus Kantam Laari, Janet Sapak, Daniel Wumbei, Issah Salifu

**Affiliations:** Department of General Nursing, University for Development Studies, Tamale, Ghana

**Keywords:** Migration, Student nurses, Intention, Healthcare

## Abstract

**Background:**

Migration among skilled labour has been noted as one of the major issues in recent times, especially among health workers. Data from the United Nations show that almost two thirds of people migrating are labor migrants and international migrants constitute 3.5% of the global migration population. Out of the millions of people who migrate across the globe, health workers, especially nurses form a greater portion of these numbers. This study explored nursing students’ intention to migrate to other countries after completing their programs.

**Method:**

A descriptive cross-sectional design approach was adopted using self-administered questionnaire that contain aspects of open-ended questions. A sample size of 226 nursing students were recruited using convenient sampling technique.

**Results:**

The results overall, revealed that 226 nursing students participated in the study. Out of this, most of the respondents 42.5% were aged between 25 and 30 years with majority 53.1% being males. Also, 35% of the participants were married with more than half 59.7% of the respondents being Christians. The results further revealed that most of the participants 64.2% had intention of migrating to other countries. Among those who intended to migrate, 11.7% identified lack of jobs, 39.3% identified low salaries in Ghana while 50.3% identified bad working conditions. The rest 2.8% attributed their intentions to migrate to educational opportunities. Common places of destination included Canada, USA, UK and Australia.

**Conclusion:**

The outcome of this study points to the urgent need for low-income countries such as Ghana to urgently put in measures to curb the menace of brain drain among nurses. Improvement in working condition of nurses must be prioritized to motivate their stay.

**Supplementary Information:**

The online version contains supplementary material available at 10.1186/s12912-024-02180-9.

## Background

According to the World Health Organization, there are an estimated 29 million nurses and 2.2 million midwives globally, with an estimated shortage of 4.5 million nurses and 0.31 million midwives forming an estimated global health workforce shortage of 4.8 million by the year 2030 [1]. Migration of health workers from one continent to another in recent years have become one of the major challenges affecting healthcare delivery globally [2]. Data from the United Nation indicates that in 2019 alone an estimated 272 million people migrated from one place to another, indicated a rise of 51 million people from the 2010 migration data [3]. The data further show that, nearly two thirds were labor migrants with international migrants comprising of 3.5 per cent of the global migration population. Out of these numbers, majority are health workforce with nurses forming a greater number of them.

Data from the United Kingdom for instance show that out of the total nurse’s population, overseas trained nurses accounted for about 18% (39,164) of the total nursing workforce [4, 5]. In the USA data show about 46% of the nurses felt overburdened during a survey with 27% of them feeling the need to leave the profession, predicting a shortage of 1.2 million for registered nurses between 2014 and 2022 [6]. Other countries including those in the Gulf such as Bahrain, Kuwait, Oman, Qatar, Saudi Arabia, and the United Arab Emirates, international nurses form as much as 79% of the total nursing workforce with most coming from countries in Africa such as Egypt [7].

Whilst shortages of nurses are acute in the African, South-East Asia and Eastern Mediterranean regions the situation is also significant in developed countries, where recent estimates suggested nine million nurse and midwifery positions remained vacant leading to recruitment from less developed countries [8].

In low- and middle-income countries such as those in Sub-Saharan Africa and Asia, shortage of health personnel such as nurses have been attributed to many factors including; inadequate education and training capacity, negative work environments, weak human resources regulatory and management systems, and inadequate financial and non-financial incentives [9, 10]. However, in recent times migration of nurses from these poor continents to already developed countries have become one of the major contributory factors to the shortage of nurses. For instance, data show that recruitment of nurses from predominately low and middle-income countries to high-income countries with developed health care systems was on the ascendency in recent times [11, 12].

The movement of nurses from less developed countries to developed countries have been influenced by several factors often termed as pull and push factors. Among these push factors mostly in their country of practice include low salaries; bad working conditions; poor management and weakness of the authorities in employing work force in the nursing profession [13]. Pull factors mostly enticing health workforce to move to developed countries include; improved and better working conditions for them and their families, clear career pathway to achieving goals, good salary schemes, demand for their services and rapid technological development in the delivery of healthcare [14].

Ghana’s health sector has been encountering challenges with the number of health workers in the country as against the population and this puts a lot of pressure on the health workers most especially nurses and doctors. Nurses migrating from Ghana have been a cause of concern for over two decades now [15]. Majority of the nurses from Ghana are migrating to the United Kingdom, United States and Canada [16]. Anecdotal figures show that about 3,000 nurses and midwives left Ghana within the first quarter of 2022 for greener pastures outside the Country [17].

These movement of nurses outside the country are motivated by push factors in their homelands and pull factors in receiving countries. This has led to some poor or developing countries losing their skilled professionals to developed countries to be concerned about the issue of brain drain. Global migration of health professionals is one of the most broadly studied issues in healthcare worldwide [18]. Challenged by a global shortage of health professionals, this movement is considered a crisis in the health setting of human resources [19].

While challenges of staffing among nurses have been a challenge in Ghana, data from the Ghana Health Service (2023) showed a nurses to patient ratio of 1:18, exceeding the World Health Organization recommendation for 1:1000 population due to significant increases in enrolment in Colleges of Nurses Training and Universities [20]. These gains made by the Government of Ghana in terms of nurse-to-patient ratio have been eroded in recent times by the increasing migration of nurses to developed countries including the United Kingdom and the USA.

Studies on nurse migration have revealed several reasons why nurses migrate from Ghana. For instance, a study among others found among others; salary differentials, established networks in destination countries, career progression paths and securing better education for their children as factors influencing migration [21]. Salary differentials, job-related factors and academic purposes were significant contribution to intention of health workers to migrate to developed countries [22].

Despite government effort to curb the increasing trend in migration among health workers including placing a ban on clearance at the ministry level for nurses, increasing the verification fees for nurses license at the Nurses and Midwives Council by 500%, adjusting salaries to serve as incentives to nurses and increasing employment levels among nurses and midwives, including those yet under training, not much have improved regarding migration among nurses. The problem has been compounded by the increasing high nurse unemployment rates due to failure of government in recent times to employ nurses because of deep economic crisis. It is upon this reflection that this study was designed to ascertain the migration intention among student nurses still under training at the University for Development Studies in Northern Ghana.

### Aim of the study

This study explored nursing students’ intention to migrate to other countries after completing their programs.

### Significance of the study

The migration of health personnel especially nurses has been a cause of concern for the past decades due its effect on the healthcare delivery of the country. This research provides information on the migration intension of student nurses. The information provided will prompt policy makers to formulate favorable policies to help retain nurses in the country. The findings have shown the common push and pull factors motivating student nurses to want to migrate after completing their program of study hence, will help health authorities better understand the phenomenon and find solutions to the menace. The study also serves as baseline information for future researches to depend on.

## Methodology

### Study design

Design of research studies are important in ensuring reliable or trusted research outcomes [23]. Research designs are informed by the type of research, purpose and objectives set for the study and the approach to participants involvement. While there are different research designs, this study adopted a cross sectional descriptive approach. This design was adopted because it is relatively cheap, less time-consuming and allows the researcher collect the responses from a larger pool and analyze at the same time.

### Study population

The population for this research were final year nursing students of the University for Development Studies. The total population of final nursing students was 520 students. The inclusion criteria involved nursing students who were aged 18 years and above. It also included student nurses who were in their final year of the course and were willing to participate in the study.

### Sample size and technique

A sample size of 226 students was arrived at for the study using Yamane formular for sample size determination to estimate out of the student population size of 520. A convenient sampling technique was used to recruit participants for the study. This approach involved the researcher moving to the individual residents of the students and administering the questionnaires to students who were qualified based on the inclusion and exclusion criteria. While this approach has biases including limitation in the chances of every student getting the chance of involvement, this approach was adopted because the period of data collection coincided with period students were on revision and so most of them were not attending classes and were staying in their private hostels.

### Data collection instrument

The instrument used in this study was developed and used by Abuosi et al. (2015). It was divided into four sections; A, B, C and D. The first section consisted of questions on socio-demographic data of respondents. The second section gathered data on migration intention of nursing students. The third section gathered data on factors influencing the decision of student nurses to migrate and the last section also gathered data on common places student nurses intended migrating to.

### Data collection procedure

The researchers after obtaining ethical approval to conduct the study met with the Head of Department of Nursing of the University for Development Studies where the purpose of the study was explained and permission sought. Individual students who were qualified to participate were contacted and those who agreed to participate after the purpose of the study was explained were made to sign consent of participation. A self-administered questionnaire was administered to the respondents who were available and willing to participate in the study. Enough time was allocated for the questionnaire to be answered by the respondents after which answered questionnaire was collected.

### Validity and reliability

Reliability and Validity are measures that are used to ensure the study is measuring the right variables in the study objectives and that same results are obtained whenever the research is replicated [24]. Measures were employed in this study in order to ensure that the right variables were used for the study and that the same results could be obtained consistently anytime the research was repeated. Reliability was ensured in this study by allowing respondents answer the questionnaires independently in order to minimize biases. Validity was also ensured in this study by ensuring questions on the questionnaire was related to the topic and objectives of the study. Steps were taken to ensure accuracy of the measuring instruments. An adapted questionnaire from Abuosi et al. (2015) was also used.

### Data analysis

The questionnaires administered were taken from the respondents after they were filled. Data was compiled and entered into the Statistical Package for Social Sciences software (SPSS) version 25 for analysis. Results from the processed data was interpreted, summarized and displayed using descriptive statistics tools such as frequency tables and charts. These tools presented the result in a meaningful manner by expressing it in percentage and numerical values.

## Results

### Demographic characteristics

Table 1is a frequency distribution table showing results on the demographic features of respondents in the study. Overall, 226 final year nursing students participated in the study with majority of them, 42.5% aged between 25 and 30 years, 30.5% between the age of 18–24 years and the least, 5.8% between the ages of 36 & above. Majority of the students, 53.1% were males with most of them, 64.2% being married. Although most of the respondents were married, only 35.4% of the married were having children. The results further showed that majority of the participants 59.7% were Christians with Muslims forming 39.4% of them. On choosing the course, most of the respondents, 55.3% chose to pursue nursing out of passion to serve. (Ref Table 1)

**Table 1:** Demographic data

Demography		Freq. (*n*)	Per. (%)
	18–24	69	30.5
Age of Respondents	25–30	96	42.5
	31–35	48	21.2
	36 & above	13	5.8
Sex of Respondents	Male	120	53.1
	Female	106	46.9
	Single	145	64.2
Marital Status	Married	79	35.0
	Others	2	0.9
Do you have any children	Yes	80	35.4
	No	146	64.6
What Religion do you belong to	Christian	135	59.7
	Muslim	89	39.4
	Traditional	2	0.9
Which Level are you in now?	Level 200 Top-Up	121	53.5
	Level 400	105	46.5
	**Total**	**226**	**100.0**

Source: Field Work, 2022

### Migration intention of nursing students

**Fig. 1:**
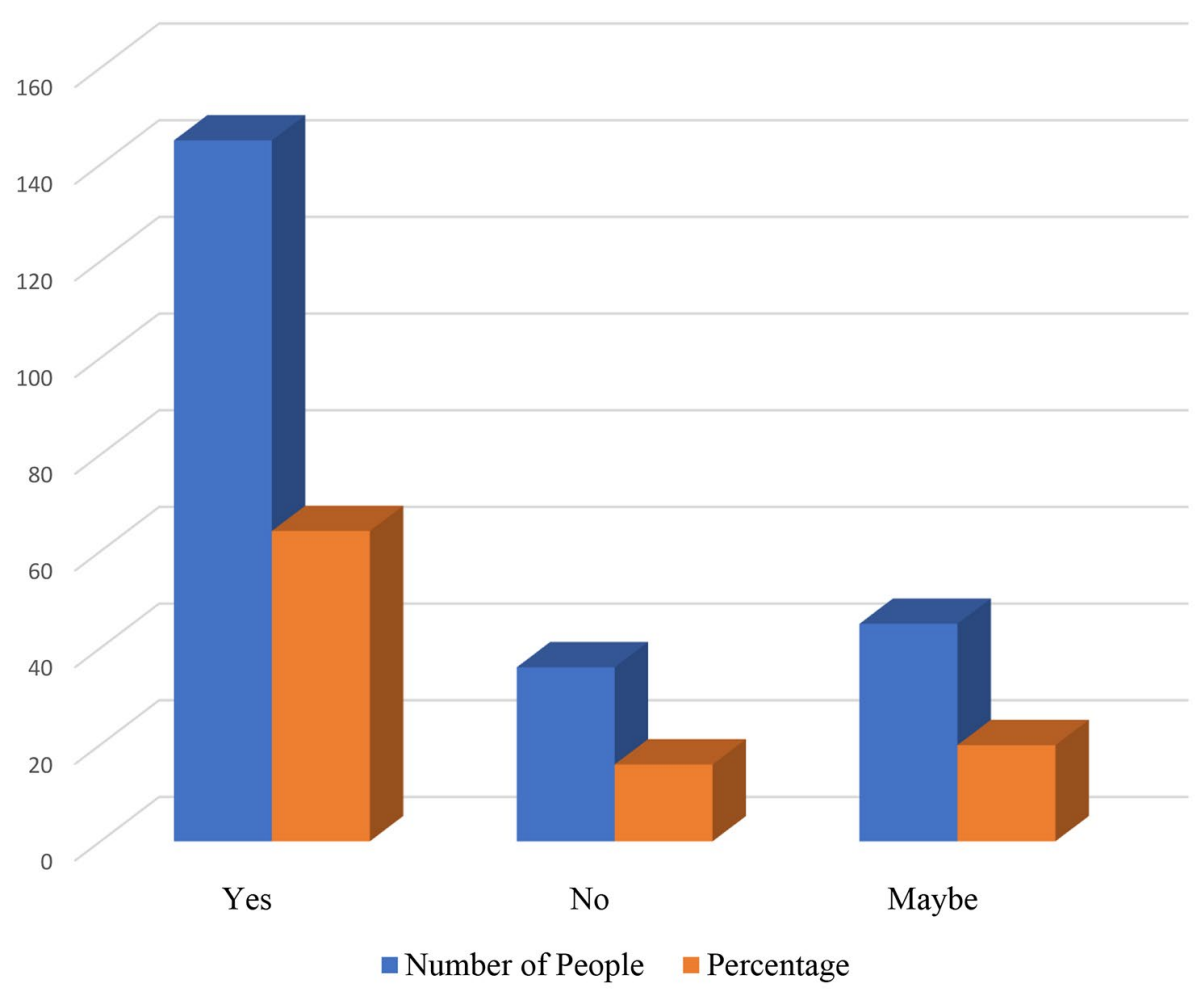
Migration intentions

This section of the study explored the intention of student nurses to emigrate after completing their program. The results revealed that most of the respondents 141(62.4%) did not feel proud practicing nursing in Ghana, with about 64.2% indicating they have the intention to leave the country after their completion of study. Pressure from significant others (15.5%), poor salaries earned by nurses in Ghana (92.9%) and poor renumeration of nurses in Ghana (46.0%) was seen as factors contributing to their intentions to migrate. (Ref: Fig. 1)

### Factors influencing nurses intentions to migrate to other countries

Respondents who intended to move out of the country were asked on factors influencing their intention to migrate to other countries. The results revealed that out of the 145 (64.2%) student nurses who intended to migrate after their program, 11.7% of the respondents identified lack of jobs as the reason they would want to travel, 39.3% based their reason of migrating out of the country to low salaries in Ghana while 50.3% want to leave because of bad working conditions. The rest of the respondents 2.8% attributed their intentions to migrate to factors including advanced technology and educational opportunities in the foreign countries. Pull factors in other countries that influenced their decision to migrate included good working conditions (32.3%), better incomes (25.2%), job availability (7.5%), with the rest indicating other attractions.

### The common places of destination

**Fig. 2:**
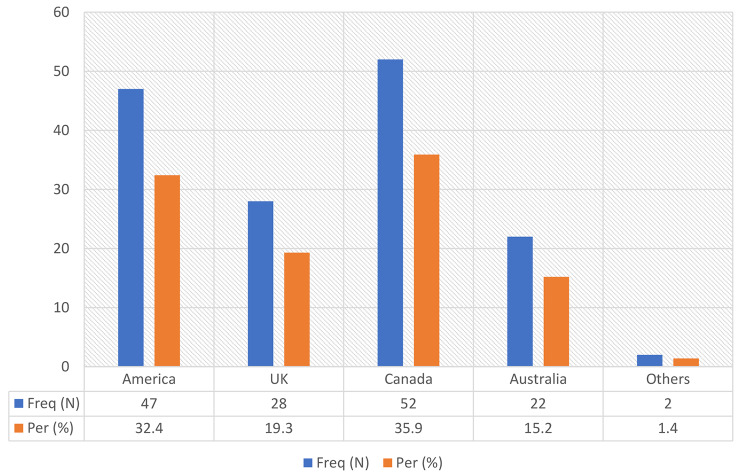
Preferred destinations

This section of the study inquired from students the place they would want to travel to if they had the chance to travel to. The results revealed that majority 35.9% of the participants would migrate to Canada, 32.4% would migrate to the United State of America, while 19.3% would migrate to the United Kingdom. The rest of the respondents 15.2% and 1.4% respectively intended to migrate to Australia and other countries such as Korea, Japan and China. (Ref: Fig. 2)

## Discussion

The results in this study in relation to the demographic findings confirms the findings made in the Ghana Statistical Service that indicated a higher number of Christians than Muslims in Ghana. The results in this study also however was not in line with the Ghana Statistical Service findings in terms of sex which indicates more females than males in Ghana [25]. The results in this study indicates more male participants as compared to several previous studies including that of one conducted by Öncü et al. (2021) where out of a total of 1,410 participants, 74.3% (1,047) were females and 25.7% (363) were males.

### Migration intention of nursing students

The result on migration intention found that most of the respondents, 64.2% had intention of migrating to work in other countries after completing their program. The findings of this study are in line with those made in a study conducted in Serbia on migration where it revealed that 69.7% of the respondents intended to move out of Serbia to work abroad [26]. The similarity in findings observed in these studies could be related to the fact that these studies were both contacted among students who are always desiring to achieve or get employed immediately after school. Both studies were also conducted using predetermined questionnaires and that could have impact of the outcomes of the study and the similarities thereof. The findings however contradict findings of a study conducted in Ghana on Ghanaian Nurses’ Emigration Intentions where they found that 48.9% of the participants constituting the minority had the intentions to migrate out of the country [16].

### Factors influencing the intentions to migrate

According to this study, majority of the respondents had the intentions to leave due to certain factors. Among these factors, included bad working condition and lack of jobs among others. These findings are similar to findings made in a related study which was conducted in Turkey which also found bad working condition as a major push factor which is making nurses in Turkey have the intention of leaving the country [27]. The findings are also in line with findings made in a study conducted in Poland on factors determining migration decisions of nursing students which found that bad working condition and low income levels were the two leading push factors that were motivating the nurses in Eastern Poland to leave the country [28].

### Common places of destination

The findings in this study reveals majority of student participants would not like to practice in Ghana after their program of study. Common places most of these students wanted to travel after completing their programs included Canada, USA, UK and Australia. These findings conform to findings made in a similar study conducted on students’ migration intention which found that most students had intentions of practicing elsewhere in other countries [29]. It further confirms findings in a study conducted in South Africa where nursing students expressed their desires to migrate and work elsewhere [30].

The findings on the other hand contradict findings made in a study conducted on common places of migration among health workers where it revealed that majority of students who participated preferred migrating to Japan [31]. The findings are also contrary to findings made in a similar study conducted in Pakistan where majority preferred migrating to the United Kingdom [29]. It further contradicts findings made in a study conducted in Ghana on migration and graduate students burn out where majority of the student preferred migrating to the United States of America [32]. The similarity in findings could be associated with the fact that in studies where findings were same, both studies were conducted among final year students and in developing countries where starting life as graduate is difficult and hence, most graduating students want to migrate to developed countries.

### Unemployment

Unemployment in low and middle-income Countries (LMICs) such as Ghana has been seen as a major contributory factor to migration especially among working class people [33]. Data indicates that more than 2 million Ghanaians representing 14.7% were unemployed by the 3rd quarter of 2023 with only about 12 million employed [34]. While limited data exist on nurses unemployment rates in Ghana, recent economic crisis coupled with high inflation rates leading to freezing of jobs in the public sector which hitherto served as the major employment sector for nurses in Ghana have rendered most nurses employed [35]. The phenomenon has resulted in instances where there are thousands of nurses sitting at home unemployed in Ghana. This high rates of unemployment have been identified as one of the reasons why nurses including student nurses harbor’s intentions to migrate [36]. Overcoming the increasing rates of health professionals migrating for greener pastures requires that central governments adopt pragmatic steps including improvement in the working conditions of health workers.

### Summary of the study

This study ascertained the migration intention among nursing students of the University for Development Studies upon completion of their study. The main objective of the study was to ascertain the migration intentions among nursing students at the University for Development. Self-administered questionnaire was used to gather data using convenient sampling technique. A total of 226 student participants were used for the study. The results revealed that majority, 66.8.2% of the participants had the intention to migrate upon completion of their program of study. The study also indicates that majority, (32.3%) of the participants are attracted to other countries due to the good working conditions out there. The results also showed that majority of the participants who had the intentions of migrating to other country preferred Canada as their choice of destination.

### Strengths

This study has highlighted the brain drain phenomenon of not only nurses in Ghana but also of the intentions of student nurses yet under training. The description of factors influencing their intentions to migrate offers opportunity for measures to be taken to mitigate the phenomenon of migration among nurses. The used of appropriate design and tool ensures reliability of the outcomes of this study.

### Limitation of the study

This study has certain limitations. For instance, it was conducted among final year undergraduate nursing students in peri-urban Ghana where unemployment levels are the highest and could have influence on students’ responses. Convenient sampling approach was also used to recruit respondents because of difficulty meeting larger number of the students together. A simple data analytic approach was used which did not involve much of inferential analysis and this is a limitation to the study. The study did not also receive funding which could have helped in expanding to larger study.

### Implication of the study

The migration of health personnel especially nurses is a cause of concern for developing countries. The outcome of this study will enable policy makers to formulate favorable policies to help retain nurses in the country. It has also espoused some of the pull and push factors motivating nursing students to migrate, thereby helping school authorities plan proper measures to help motivate newly graduated nurses to stay and work in the country.

Again, it will also serve as a research document concerning migration of nurses for other researchers.

## Conclusion

The study in conclusion recognizes the fact that most nursing students in developing countries habour the intention to migrate after completing their program of study. It also recognizes that majority of the participants who intended migrating did so with the aim of seeking for better working conditions. According to the study, Canada was the most preferred choice of destination for the participants.

## Recommendations

Based on the findings of this study, the following recommendations are made to assist policy makers improve upon curbing the increasing cases of migration of nurses;

1. Governments should keep on improving the working conditions of nurses in order to retain them and to attract more people into the nursing profession.
2. Trained nurses should only be allowed to leave the country to pursue higher education after which they should be made to return using policies between governments.
3. Means of supporting the education of nurses should be put in place but should be arranged on a bond to ensure nurses stay loyal to their country.
4. More hospitals should be built to employ nurses who are yet to be posted to reduce or bring down the number of unemployed nurses.
5. Efforts should be made by governments to improve on the nurse patient ratio by employing more nurses especially in rural areas.

### Electronic supplementary material

Below is the link to the electronic supplementary material.

Supplementary Material 1

## Data Availability

Data for this research is available and has been uploaded as a supplementary file.
