# Supplementary material for: Migration intentions among nursing students in a low-middle-income country
Source: BMC Nurs. 2024 Jul 18;23:492. doi: 10.1186/s12912-024-02180-9 (PMC11256540; doi:10.1186/s12912-024-02180-9)
Supplement: Supplementary file 1 — Supplementary Material 1 [file 12912_2024_2180_MOESM1_ESM.docx]

**APPENDICES**

**QUESTIONNAIRE**

This study is on Migration Intentions of Student Nurses. Participation is purely voluntary and you can withdraw from the study but we would be much grateful if you could make time out of your busy schedules to respond to the questions below. We ensure you that any information provided would be treated confidentially and be used for only academic purposes.

Kindly select the appropriate answer or write your responses in the spaces provided where applicable.

**Section A:**

**Socio-demographic Data**

1. Age (in years)

(1) 18-24 ……………. [ ]

(2) 25-30 ……………. [ ]

(3) 31-35 ……………. [ ]

(4) 36 & Above ……………. [ ]

2. Sex:

(1) Male …………[ ] (2) Female……….[ ]

3. Marital Status:

(1) Single …. [ ] (2) Married……[ ] (3) Others……[ ]

4. Do you have any children?

(1) Yes …………. [ ] (2) No ……………[ ]

5. What religion do you belong to?

(1) Christian ……. [ ] (2) Muslim …………[ ] (3) Traditional ……[ ]

6. Which level are you in now?

(1) Level 200 Top-Up …… [ ] (2) Level 400………[ ]

7. What influenced you to choose nursing as your program of study?

(1) Salary …………… [ ]

(2) Passion to care for patients …………… [ ]

(3) Job security ………………… [ ]

(4) Desire to migrate and work outside Ghana ………………… [ ]

(5) Others (specify)…..........…………………………………………………

**Section B:**

**Intention to Migrate**

8. Do you think Ghanaian nurses' income is sufficient?

(1) Yes……[ ] (2) No ……[ ] (3) Don't know ……[ ]

9. How would you describe remuneration of nurses in Ghana?

(1) Very good…… [ ] (2) Good ……[ ] (3) Very poor………[ ] (4) Poor ……[ ]

10. How do you think others view the nursing profession?

(1) Very good…… [ ] (2)Good……[ ] (3) Bad……[ ]

11. Would you be proud to work as a nurse in Ghana?

(1) Yes……… [ ] (2) No………[ ]

12. Do have any intention to leave Ghana after your completion of study?

(1) Yes...… [ ] (2) No……[ ] (3) Maybe……[ ]

13. Are you being pressured from any person or group to practice outside Ghana?

(1) Yes…… [ ] (2) No……[ ]

14. If yes, indicate relationship with person of group

(1) Parents… [ ] (2) Siblings… [ ] (3) Spouse… [ ] (4) Friends… [ ] (5) Others… [ ]

**Section C**

**Factors of Migration Intention**

15. Would you like to practice in Ghana after your program of study?

(1) Yes……… [ ] (2) No………[ ]

16. Reasons to either practice in Ghana or outside Ghana

(1) Low salary… [ ] (2) Poor conditions of service… [ ] (3) Inadequate jobs… [ ]

(4) To serve my country… [ ] (4) Can’t leave my family… [ ] (6) Others… [ ]

**Section D:**

**Preferred Destinations**

17. What is your preferred country of destination?

(1) Australia [ ] (2) Canada[ ]

(3) The United States of America……… [ ] (4) United Kingdom…………[ ]

(5) Others (Specify)…..............………………………...

18. What attracts you to the country of your choice?

(1) Job availabilities…… [ ] (2) Better income……[ ] (3) Good working conditions…[ ]

(4) Others (Specify)…...............................................

19. Do you wish to return to Ghana after some time?

(1) Yes…… [ ] (2) No……[ ] (3) Don't know……[ ]
